# Characterizing the Hourly Variation of Urban Heat Islands in a Snowy Climate City during Summer

**DOI:** 10.3390/ijerph16142467

**Published:** 2019-07-11

**Authors:** Chaobin Yang, Ranghu Wang, Shuwen Zhang, Caoxiang Ji, Xie Fu

**Affiliations:** 1 School of Civil and Architectural Engineering, Shandong University of Technology, Zibo 255000, China; 2 Institute of Loess Plateau, Shanxi University, Taiyuan 030006, China; 3 Northeast Institute of Geography and Agroecology, Chinese Academy of Sciences, Changchun 130102, China; 4 Shenyang Meteorological Bureau, Shenyang 110168, China; 5 School of Architecture and Urban Planning, Suzhou University of Science and Technology, Suzhou 215009, China

**Keywords:** hourly, air temperature, urban heat island intensity, land use, temporal variability

## Abstract

Temporal variation of urban heat island (UHI) intensity is one of the most important themes in UHI studies. However, fine-scale temporal variability of UHI with explicit spatial information is sparse in the literature. Based on the hourly air temperature from 195 meteorological stations during August 2015 in Changchun, China, hourly spatiotemporal patterns of UHI were mapped to explore the temporal variability and the effects of land use on the thermal environment using time series analysis, air temperature profiling, and spatial analysis. The results showed that: (1) high air temperature does not indicate strong UHI intensity. The nighttime UHI intensity (1.51 °C) was much stronger than that in the daytime (0.49 °C). (2) The urban area was the hottest during most of the day except the period from late morning to around 13:00 when there was about a 40% possibility for an “inverse UHI intensity” to appear. Paddy land was the coolest in the daytime, while woodland had the lowest temperature during the nighttime. (3) The rural area had higher warming and cooling rates than the urban area after sunrise and sunset. It appeared that 23 °C was the threshold at which the thermal characteristics of different land use types changed significantly.

## 1. Introduction

The urban heat island (UHI), which is the phenomenon where cities are typically warmer than surrounding rural areas is a result of the process that natural landscapes with vegetation and permeable surfaces are gradually replaced by dense buildings with large heat capacity [1,2,3]. Along with anthropogenic heat sources, the transformation alters the natural solar and hydrological balances of cities, thereby making UHI a common phenomenon worldwide [4,5,6,7,8]. The UHI and heat waves are thought to have a large impact on energy consumption [9], human health [10], air pollution [11], and ecological balances [12]. As the rapid urbanization and industrialization continue, more people will be influenced by UHI, and it will impose new challenges for sustainable urban planning [13]. Thus, it is necessary to have a obtain a better understanding of the UHI, which will help in making adaptions to climate change and human development [14].

UHI intensity varies constantly [15]. As a result, the temporal variation of UHI intensity has become one of the most important themes in UHI studies [16,17,18]. Currently, two efficient methods are used to conduct UHI research, namely meteorological observations [19,20] and thermal remote sensing [21,22]. Owing to the availability of remote sensing images, daily, monthly, seasonal, annual, and long-term variations of surface urban heat island (SUHI) intensity are more common in the literature [19,23,24]. However, there are two main limitations in current SUHI research. First, the validation of satellite-derived land surface temperature (LST) is difficult to conduct owing to the mismatch between resolution of images and ground measurements [25]. Second, higher temporal resolution, for example hourly variations of UHI, is not well recorded in the literature [15]. Unfortunately, available literature on this theme is sparse. SUHI studies conducted by thermal remote sensing are subject to the local time when the images are acquired. Appropriate quantification of UHI is an essential foundation for studying the effects of extreme heat [26]. 

Different from LST, air temperature is observed by precise instruments in weather stations with high accuracy and consistency. In addition, air temperature is closer to the apparent temperature, which affects residents’ behaviors and choices [26]. A reinforced understanding of the hourly variations of UHI can benefit dwellers and urban managers in many ways. For example, it can help dwellers to manage their time for outdoor activities in daily life and may reduce energy consumption by decreasing the utility time of air conditioning [27,28]. In the past, limited meteorological stations or the use of automobile transects made it difficult to conduct spatially continuous analysis. Nowadays, a large air temperature network with sufficient weather stations has overcome the limitations of spatial resolution. For example, a dense meteorological network of 23 locations across 5 y were used to monitor the urban climate in a small Portuguese city and adequate results were obtained [29]. With the advantages of high temporal resolution and accuracy, meteorological observations can provide fine-scale temporal information on UHI.

Numerous studies have explored the effects of different land use types and surface factors on the SUHI [17,30,31,32]. For instance, a significant correlation was found between impervious surfaces and LST [33,34], while vegetation and water bodies could effectively reduce the SUHI intensity [35,36,37,38]. Most of these studies employed LST to explore the relationships between the SUHI and land use. As mentioned previously, the results of the influence of land use on the SUHI were based on a particular time. The cooling and warming rates of urban and rural areas should also be discussed UHI studies with high temporal resolution [20]. In addition, the effect of the urban canopy and radiation balance on the causes of UHI have been explored by many studies [39,40,41]. However, the fine-scale variations of air temperature among different land use types are seldom reported. In other words, there is little literature about how the air temperature of different land use changes during a day at different local times. As a result, it is very difficult to comprehensively determine the hourly variation of temperature among land use types in a comprehensive way using LST/RS. 

The UHI effect differs by city. The UHI intensity of cities located in tropical and temperate zones has been explored in many studies [7,19,42,43,44]. However, information on UHI intensity characterized by air temperature in snowy climate cities needs to be strengthened to enrich the theoretical and case studies. Taking Changchun, which is a cold zone city with hot summers (June-August), as a case study, hourly air temperatures collected from 195 meteorological stations during August 1-31, 2015 were used to explore the hourly evolution of the UHI. The specific objectives were to (1) investigate the hourly variations of air temperature and UHI intensity to determine their differences and (2) analyze the air temperature differences among land use types to explore their different hourly cooling and warming rates. It is hoped that this study will enhance our understanding of the hourly variations of UHI and provide insights into UHI mitigation measures to benefit planners, decision makers, and dwellers.

## 2. Materials and Methods

### 2.1. Study Area

**Figure 1:**
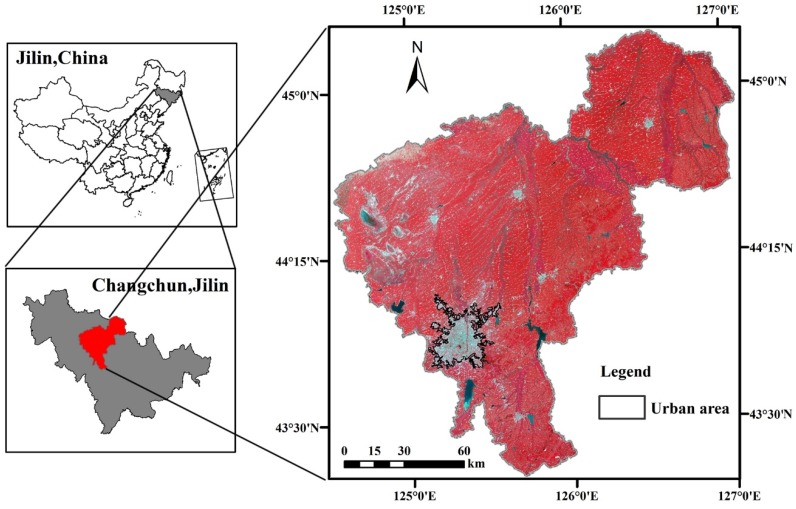
Location of the study area of the municipality Changchun (Landsat8 false-color images).

Changchun (43°05′–45°15′N, 124°18′–127°05′E, Figure 1), which is the capital of Jilin Province, is situated at the heart of the Songliao Plains in northeast China; it had, in an area of 20,593.5 km^2^, a population of approximately 7.53 million in 2016. It serves as the economic, cultural, and political heart of the province. The city is 250–350 m above sea level. Changchun is characterized by a sub-humid continental climate with a windy spring, a short, hot summer, an autumn with large diurnal temperature variation, and a long, cold winter. Based on the local meteorological records, the annual average precipitation and air temperature of Changchun is 561 mm and 5.5 °C, respectively. The landscape of Changchun is a mosaic of urban areas, paddy land, dry land, woodland, water, rural settlements, other built-up areas and unused land. In the past 30 years, dramatic changes have taken place in the patterns of land use and land cover changes (LUCC), which have a significant influence on the thermal environment [24].

### 2.2. Data Sources

Hourly air temperature data during August 1–31, 2015 were obtained from the Changchun Air Temperature network, which consists of 195 scattered sites, to characterize the thermal environment. These weather stations were operated by the Meteorological Bureau of Changchun city, which was part of the National Meteorological Information Center of the China Meteorological Administration (CMA). All the data were collected by sensors placed inside instrument shelters at a height of 1.5 m above the ground surface. All the instrument shelters were installed above natural surfaces (typical grassland) and away from artificial buildings. Because of ventilation and the avoidance from sunlight, the air temperature data had adequate representativeness. However, the observed data were easily influenced by geography and topography, so data quality control (DQC) was performed by CMA [45]. Based on the hourly records of the Kuancheng weather station (43.81° N, 125.31° E), during August 2015, the weather in Changchun was mostly sunny, and the total precipitation, most of which was from thundershowers, was 117.4mm, which was distributed in 30 h out of 744 (24 h × 31 d) h (4%). Of the 195 observation sites, 24 were in the urban area and 171 were in the rural area. In total, more than 140,000 hourly air temperature data points were obtained in this study. After DQC, the accuracy of these data provided by the CMA was close to 100% (http://www.cma.gov.cn). Taking 31 d of 24 h data collected from 195 meteorological stations into account, this study produced more robust results. According to the local weather station records, the average sunrise and sunset times for Changchun in August 2015 were before 05:00 am and after 18:00, respectively. Thus, in this study, the daytime was from 05:00 to 19:00 (14 h), and the nighttime was from 19:00 to 05:00 (10 h) in the Changchun area.

The newly launched Landsat 8 in 2013 extended the capabilities for mapping and monitoring of land cover and other land surface features by at least 5 y [46]. Landsat 8 images with good quality (clear atmospheric conditions), which were acquired on August 29, 2014 (path 118/row29, path 118/row30) and September 24, 2014 (path 119/row 29) from the United States Geological Survey, were used to extract land use information.

### 2.3. Methods

#### 2.3.1. Land Use Information Extraction

Pre-processing included radiometric correction and co-registration. Bands 5, 4, and 3 of the Landsat 8 images were used to create false-color images. Then, according to the natural environment of Changchun and prior knowledge, visual interpretation was employed to extract the information of eight land use/land cover types, including urban area, paddy land, dry land, woodland, water, rural settlements, other built-up area, and unused land based on their color, location, shape, and size [24,47]. Google Earth, high spatial resolution images GF-1, digital elevation model, and data on soil and vegetation type served as auxiliary data to help identify the land use types. According to the fieldwork conducted during August 2015, the total accuracy of the land use map was over 91.6% [24].

#### 2.3.2. Temporal Variations of UHI Intensity

UHI intensity refers to the air temperature difference between the urban area and that measured in rural areas. According to the definition of UHI intensity, it could be calculated as follows:

(1) $$UHI\;intensity=T_{urban}-T_{rural}$$

where $T_{urban}$ is the hourly average temperature of urban areas and $T_{rural}$ is the average temperature of rural areas. $T_{urban}$ and $T_{rural}$ were calculated as follows:

(2) $$T_{urban}=\sum_{1}^{i}T_{urban\left(i\right)}/i$$

(3) $$T_{rural}=\sum_{1}^{j}T_{rural\left(j\right)}/j$$

where $T_{urban\left(i\right)}$ and $T_{rural\left(j\right)}$ are the hourly mean air temperatures of single meteorological station located in urban and rural areas, respectively. In addition, *i* (*24*) and *j* (*171*) are the numbers of weather stations located in the urban area and rural area, respectively.

For single meteorological stations, the hourly mean air temperature $T_{h}$ was calculated as follows:

(4) $$T_{h}=\sum_{1}^{n}T_{h\left(n\right)}/n$$

where *h* (*0,1,…23*) is the local time of day, and *n* (*1,2,…31*) is the date in August 2015.

Time series (hourly) from 00:00 to 23:00 (local time) were used to analyze the temporal variability of UHI intensity and the air temperature for urban, rural, the whole study area and some typical weather station samples.

**Figure 2:**
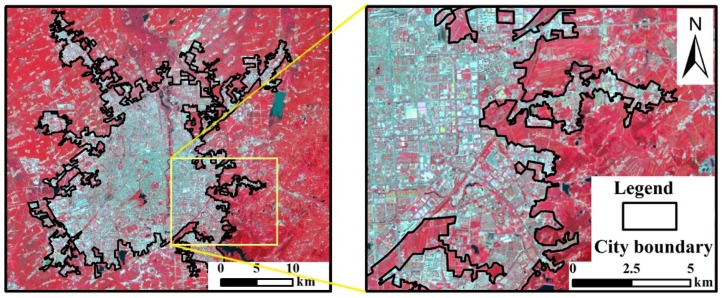
The identification of urban area in this study.

One important point that requires more explanation is the definition of urban and rural area. In Wu’s review article, high human population density and extensive impervious surface area are two most widely used factors to define urban areas [48]. According to the thermal differences between man-made land cover and natural surfaces, the city boundary was identified by visual interpretation mainly based on the percentage cover of impervious surfaces. The urban area is a continuous area without interruption in space. The urban area in this study is shown in Figure 2. 

#### 2.3.3. Characterizing the Thermal Environment

The availability of spatially explicit information on air temperature is important for policy and decision making. In order to explore the spatiotemporal patterns of the thermal environment, hourly air temperature maps of the whole study area were created by interpolating the data from 195 weather stations. Inverse distance weighted interpolation, determining cell values using a linearly weighted combination of a set of sample points, was used to interpolate the air temperature into 30m grid maps, which was consistent with the resolution of LUCC datasets [49,50].

According to the values of the interpolated maps, the study area was reclassified into seven different regions: highest, higher, high, medium, low, lower, and lowest, to depict the thermal environment (Table 1) [24,51]. In this study, zones belonging to high, higher and highest were treated as H-region, which was warmer than other zones, and zones belonging to low, lower, and lowest were treated as the L-region.

#### 2.3.4. Air Temperature Profiling

The profiling approach is widely used to show the temperature differences across space in UHI studies [33]. Temperature profiles across urban and rural areas can have considerable fluctuations, such as several spikes, basins, valleys, and plateaus [24,52]. The fluctuations in the temperature profile can provide a spatially explicit temperature gradient in the rural-urban-rural transection.

In this study, based on the principle that the profile should be across different land use types and pass through the whole study area, a southwest-northeast profile was produced according to the means of hourly interpreted air temperature maps. Four weather stations that were located nearest to the profile line were selected as sample points to better provide transection information. As a result, 24 profiles representing 24 h were used explore the spatiotemporal variations at the same time.

#### 2.3.5. Spatial and Statistical Analysis

The hourly mean air temperatures of eight different land use types were calculated by overlay and zonal spatial statistics analysis to investigate its hourly variations based on the interpolated maps. Then, the land use temperature difference (*LUTD*) was defined as follows:

(5) $$LUTD_{i}=T_{max}-T_{i}$$

where $T_{max}$ is the maximum temperature among the land use types and $T_{i}$ is the temperature of land use type *i*. Total *LUTD* is the sum of all the $LUTD_{i}$. It can be used to characterize the temperature differences among land use types. Taking air temperature as the independent variable, and total LUTD as the dependent variable, bivariate correlations and scatter plots with linear regressive models were created to determine the influence of air temperature on $LUTD_{i}$. All the statistical analyses were conducted using SPSS 19.0 in this study.

To explore the relationships between land use types and air temperature, we computed the mean hourly, daily, daytime, and nighttime and the temperature difference between the daytime and nighttime (TDDN) for eight land use types in this study. 

## 3. Results

### 3.1. Spatial Pattern of Land Use

**Figure 3:**
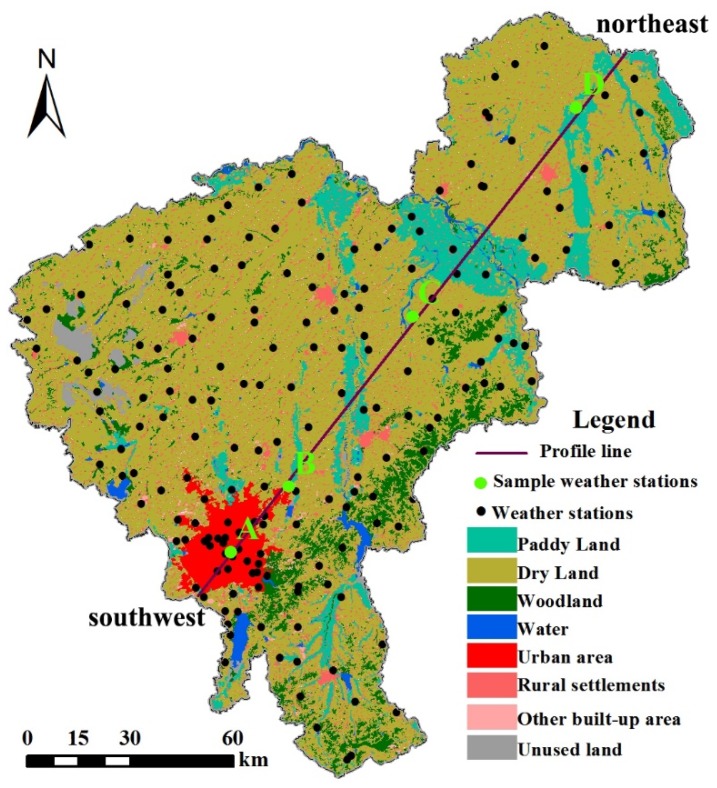
The southwest-northeast profile and the spatial pattern of land use.

Figure 3 shows the spatial pattern of land use in this study area, and Table 2 shows the area percentage of different land use types. Based on the classification results, it was found that urban area only occupied 2.80% of the whole area, but there were 24 weather stations (12.31%) in the urban area. Dry land had the largest area occupying almost 70%. Woodland and rural settlements covered 7.16% and 7.56%, respectively, while the area of water, unused land, and other built-up area were relatively small.

### 3.2. Hourly Variations of Air Temperature and UHI Intensity

**Figure 4:**
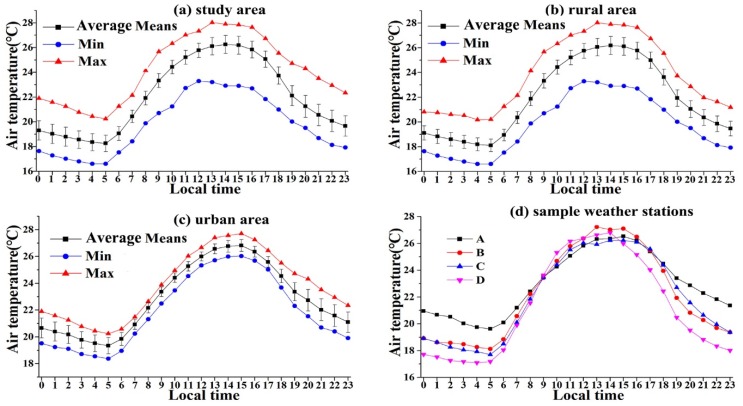
Hourly variations of maximum (Max), minimum (Min) and mean air temperatures of study area, rural area, urban area, and the averaged mean air temperature of four sample weather stations.

Hourly variations in the mean with standard deviation, maximum and minimum air temperature for the study area, rural area, and urban area, and the average air temperature for four representative weather stations are shown in Figure 4. For the overall trends of the air temperature, all the different areas and weather stations showed similar trends in that the air temperature decreased from 00:00 to around 05: 00, then increased until around 14:00, and then decreased again. The trend looked like a horizontal “S-shape”.

The urban area had a higher maximum hourly, minimum hourly, daytime, nighttime, and mean hourly air temperature compared to those of the rural area (Table 3). The maximum recorded temperature and the time when it was obtained varied between the urban and rural areas. The highest and lowest hourly air temperatures for the urban area were around 26.81°C at 15:00 and 19.27°C at 05:00, respectively. Meanwhile, for the rural area, they were 26.18°C at 14:00 and 18.08°C at 05:00, respectively. Based on the records of the local meteorological bureau, the time of the sunrise in Changchun during August 2015 was from 04:26 to 05:00. The lowest air temperature for both the urban and rural area occurred around sunrise.

**Figure 5:**
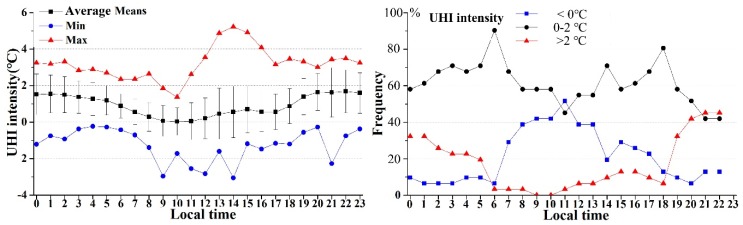
The average means hourly variations of UHI intensity and the hourly frequency distribution of different UHI intensity for the period of August 1–31 in Changchun city.

The hourly variations of UHI intensity and its hourly frequency during August 2015 are shown in Figure 5. The mean values with standard deviations, maximum, and minimum for each hour of the day are also shown. Different from the horizontal S-shaped trend in air temperature, the trend of variation in UHI intensity looked like an elongated “V”; this was consistent with previous studies [19,53], but was not expected as the rainy season in a tropical city of northeastern Brazil [54].

The range in mean hourly UHI intensity for the whole area was from a minimum of 0.03 °C at 10:00 to a maximum of 1.64 °C at 22:00 (Table 4). The time when the maximum (22:00) and minimum (10:00) UHI intensity appeared was very different from the time when the maximum (around 14:00 and 15:00) and minimum (05:00) air temperature occurred. 

The values of UHI intensity ranged from −2.97 °C to 5.22 °C. The higher UHI intensity usually occurred during the nighttime. During the nighttime, more than 20% of the days showed an UHI intensity greater than 2 °C, and between 20:00 to 22:00, the proportion reached over 40%. An UHI intensity over ± 2 °C was rare during the daytime because the temperature of the urban area and rural area was very similar. This finding was expected, as studies conducted in Orlando [20]. The UHI intensity declined quickly after sunrise and reached its lowest value (nearly 0 °C) during 09:00 to 11:00. On average, most of the UHI intensity values were between 0 °C and 2 °C.

Table 4 shows the mean hourly UHI intensity values of the whole study area. The mean UHI intensity for the whole day was 0.92 °C, but the average night UHI intensity (1.51 °C) was much higher than that of the daytime. 

**Figure 6:**
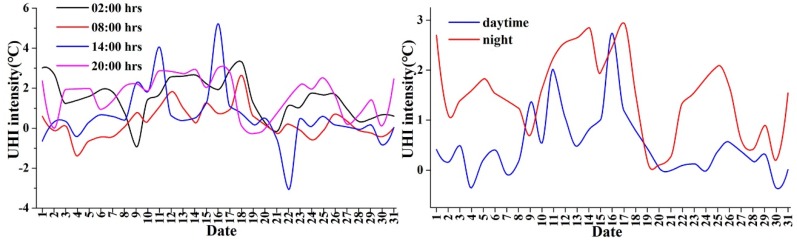
The diurnal variations of UHI intensity at representative hours and in the daytime and nighttime during August 1–31, 2015.

Although Figure 5 and Table 4 show the mean hourly variations in UHI intensity, we explored the diurnal variations among each day during August 1–31, 2015 (Figure 6). For most days, the UHI intensity values at 02:00 and 20:00 were higher than those at other time. However, the range of UHI intensity at 14:00 was the largest. 

It seemed that the UHI intensity showed larger variations during the hottest period (13:00–15:00) (also seen in Figure 5). For the difference in UHI intensity between the daytime and nighttime (Figure 6), it could be seen that the daytime UHI intensity was rarely larger than that of the nighttime [55].

### 3.3. Spatiotemporal Pattern of the Thermal Environment

**Figure 7:**
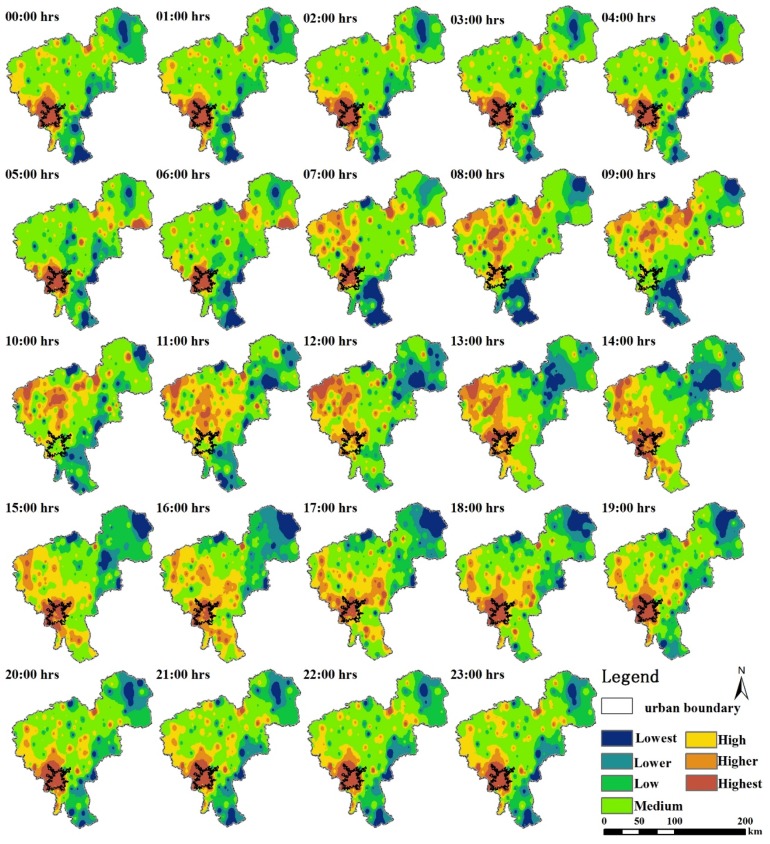
Mean hourly variations of air temperature for Changchun during August 1–31, 2015.

The air temperatures observed at 195 weather stations in Changchun made it possible to map the hourly variations of the thermal environment in a spatially explicit way by interpolation. As mentioned, in Section 2.3, hourly maps reclassified into seven air temperature regions with different colors were produced (Figure 7), thereby making it easier to distinguish hot regions.

As shown in Figure 7, the general pattern of the thermal environment in Changchun exhibited significant hourly differences. From 00:00 to 06:00, the urban area had the highest air temperature. During this period, there were few H-regions in the rural area. At 07:00, a turning point occurred. Although the urban area was still the hottest region, some higher or highest zones appeared in the northwest part of the rural area. Over time, an interesting phenomenon occurred during 08:00 to 12:00. The air temperature of some of the rural area was much higher than that in the urban area. The highest zones were in the northwest part where dry land and unused land (bare soil and saline land) were the dominant land use types. There was almost no distribution of land use types with cooling effect, such as paddy land and woodland, in this area. As a result, the UHI phenomenon was not apparent during this period. After 13:00, the urban area became the one of hottest regions again, but some rural area still had very high air temperatures at the same time. From 15:00 to 19:00, the high temperature zone with some higher zones began to appear in the central part of the study area. During 20:00 to 23:00, the city area was the hottest. The northeast and southeast parts of the study area were cooler than the rest of Changchun throughout the day.

Table 5 shows the proportion of different temperature zones and the H-region in Changchun city for 24 h. For the H-region, during the late nighttime-morning (20:00–07:00), it accounted for less than 30% of the whole area. However, the proportion of the highest zone was greater than 5.5%, which was higher than that during other times.

**Figure 8:**
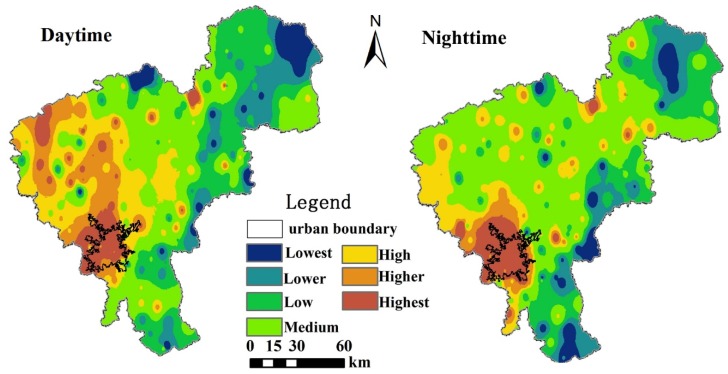
Mean daytime and nighttime air temperatures for Changchun during August 1–31, 2015.

Figure 8 shows the difference in the thermal environment between the daytime and nighttime in Changchun based on the mean air temperature maps. The largest difference was that the area of the H-region was much larger during the daytime than during the nighttime. During the daytime, the southern part of the urban area did not have the highest temperature compared to that in the nighttime. The L-region had a similar spatial distribution in both the daytime and nighttime. It appeared that the H-region was more sensitive to changes in temperature than the L-region.

### 3.4. Profile of Air Temperature

**Figure 9:**
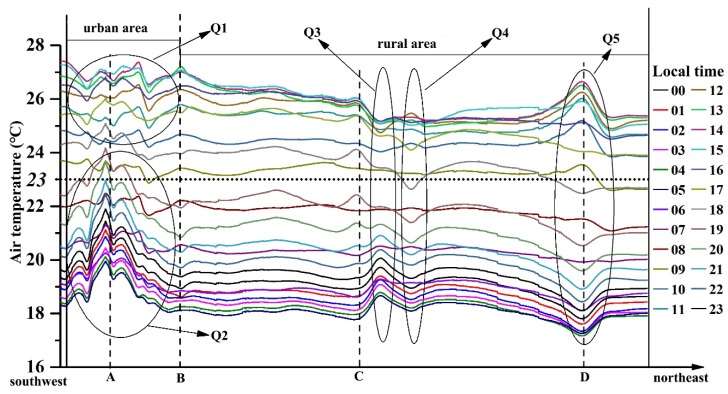
Air temperature profiles over 24 h in the direction from southwest to northeast (A, B, C and D are the sample weather stations, and their locations are shown in Figure 3).

The spatial information of the four selected weather stations was as follows: A was in the downtown area, B was located in the fringe area between the urban and rural area, C was located in the dry land, and D was surrounded by paddy land (Figure 3). The air temperature profiles showed that in the direction from southwest to northeast, there was an UHI phenomenon where urban area had higher air temperatures (Figure 9). 

Along the urban-rural gradient, the air temperature decreased from the city center to the rural areas. However, several fluctuations were also found in the profiles, and Q1–Q5 were five clusters that attracted our interest (shown in Figure 9). In Q1, the distribution of air temperature in the urban area did not show regular patterns which generated counterintuitive trends. Meanwhile, in Q2, the city center had much higher temperatures than the surrounding areas, which was consistent with local knowledge. In Q3, a weather station was located in the rural settlements. Peaks appeared in Q3 when the air temperature was lower than 23 °C, while there were several valleys when temperature was higher than 23 °C. A temperature of 23 °C meant that the air temperature of dry land was 23 °C. Q4 was a paddy land area, and the trend of change in air temperature was opposite to that of Q3. Q5 was in dry land, but it was surrounded by a large area of paddy land. As a result, the air temperature in this area was affected by the combination of dry land and paddy land.

### 3.5. The Air Temperature Difference among Land Use Types

**Figure 10:**
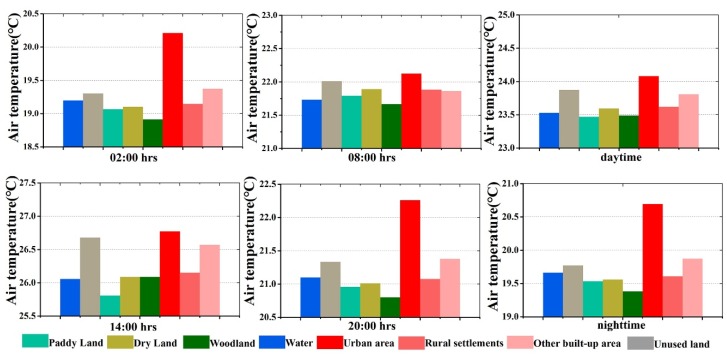
The air temperature among land use types at different times.

As shown in Table 6, the temperature of different land use types showed clear differences. The urban area had the highest temperature in the nighttime, daytime, and whole day, but had the smallest TDDN. The woodlands had the lowest temperature in the nighttime and whole day, while the mean temperature of the paddy land during the daytime was the lowest. The woodlands together with unused land had the largest TDDN. For hourly variations, the urban area had the largest values during most hours, except from 09:00 to 12:00, during which the unused land mainly located in the northwest part of the study area was the hottest. As a result, the UHI intensity was close to zero during this period. On the contrary, the woodlands had the lowest temperature, except for from 12:00 to sunset, which was when paddy land was the coolest. To show the temperature difference among the different land use types more clearly, Figure 10 shows the differences at four representative hours, daytime, and nighttime with the same temperature scale in the y axis. It can be seen that at 02:00, 20:00, and nighttime, the temperature of the urban area was much higher than that of the other land use types. Meanwhile, at 08:00 and in the daytime, the temperature differences between the urban area and rural land use types were not significant. At 14:00, the temperatures of unused land and other built-up area were much higher than those of the other rural ones, and were almost as high as that in the urban area.

## 4. Discussion

### 4.1. The Temporal Features of UHI Intensity

A better understanding of the fine temporal features of UHI intensity during the daytime could influence the suggested time for outdoor activities, preparation for the energy use of air conditioners and identification of the time when disease related to heat waves occur [56,57].

It was found in this study that the hourly variation of urban air temperature was very different from that of UHI intensity. High temperatures in a city do not indicate intense UHI intensity. In addition, during 08:00 and 13:00, approximately 40% of the days between August 1 and August 31 showed an “inverse” UHI intensity. This may have been due to the fact that there was a large area of bare soil with high air temperatures outside the urban area. For daytime and nighttime differences, Changchun exhibited a prominent nocturnal UHI effect with an average intensity of 1.51 °C, and the mean daytime UHI intensity was 0.49 °C. The phenomenon that nocturnal UHI intensity was stronger than that in the daytime was consistent with other studies [58], even although Changchun is a snowy climate city. 

UHI intensity is the temperature difference between urban and rural areas. It can be affected by external factors, such as solar radiation, wind speed, cloud cover, location, time of day, and season, and internal factors, such as the different materials in urban and rural areas and their sensitivities to the environment [3,5,59]. For temporal variations, solar power plays an important role in the thermal environment [60]. The difference in solar energy received by the Earth at different times is the most important driving force. During the nighttime, there is no solar energy, and the temperature is related to the latent heat flux [21]. 

### 4.2. The Spatial Features of Air Temperatures

The spatial patterns of land use with different thermal characteristics can have an important influence on the distribution of the thermal environment [24]. In general, the urban area was warmer than the rural area most of the time during the hottest season, especially at nighttime. However, it was apparent that the thermal environment also varied significantly in different areas and at different hours (Figure 4 and Figure 7). For spatial variations, there was a large difference between the urban area which consisted of mass asphalt, concrete, metal and glass that affected the thermal environment, and rural natural landscapes, such as forests and crops. Land use types with different thermal characteristics according to the changes of temperature explained the spatiotemporal variations to some extent, as also reported by Zhang [61].

The mean temperatures of some of the rural stations were higher than those at station A during this period (Figure 4d). One possible reason for this was that station A was located in a green space or other cooling land use types (parks), which had low temperatures in the city area. Another possible explanation was that the air temperature of some land use types, such as unused land (bare soil and sand), was high during the daytime. 

Compared to the urban area, the rural area had a larger temperature range. For some bare land, the temperature could have been higher than that in the city; meanwhile, there was a large amount of woodland and paddy land in rural areas whose temperatures were always lower than those in the urban area. In addition, the higher latitude in the northeast part with less solar energy may have also caused low temperatures. 

The cause of the higher air temperature in the city area could have been partially explained by mass anthropogenic heat, heat stored and re-radiated by urban buildings with high heat flux [62], and lack of green spaces. However, as described in Section 3.4, when the air temperature of dry land was higher than 23 °C, it seemed that the temperature differences between the urban and rural areas were small and the urban area did not show regular patterns. It could be inferred that 23 °C was the threshold in this case as the air temperature profiles showed different trends. The UHI intensity would not have been severe when the temperature was higher than 23 °C. One possible reason for this was that the land use was not sensitive to high temperature when the air temperature was higher than 23 °C. 

### 4.3. The Warming and Cooling Rates of Different Land Use

Different land use types usually have different thermal variations and heat capacities. As mentioned, the daytime UHI intensity was much weaker than that of the nighttime, and began to decline quickly after sunrise. 

When we examined the warming rates around sunrise (05:00), as expected, the urban area had the smallest warming rate of 0.62 °C/h from 05:00 to 06:00. Meanwhile, the warming rate of rural land use at this time was greater than 0.74 °C/h with the largest value of 0.9 °C/h for unused land. In the next four hours (from 06:00 to 10:00), all the land use types had very high warming rates of over 1.20 °C/h. During this period, from highest to lowest, the warming rates of unused land, dry land, rural settlements, paddy, woodland, other built-up area and urban area were 1.39, 1.37, 1.37, 1.35, 1.35, 1.34, 1.33 and 1.22 °C/h, respectively. During this period, warming rates in the rural area were larger than those in the urban areas, thereby meaning that urban area was not the hottest zone. After 10:00, the urban area again had higher warming rates than rural areas until 15:00 when their temperatures became to decline. After sunset, the variations of cooling rates were different from warming rates. Although the cooling rates at both the urban and rural sites were greater around sunset than during other times, the cooling rates of the urban area was much smaller than those of the rural areas. As a result, the temperature of the rural areas declined sharply. In particular, the cooling rates of the woodland from 18:00 to 19:00 reached as high as −1.77 °C/h. 

For the rural areas, the temperature declined quickly without energy input after sunset. For the urban area, owing to the large building heat capacity, heat emitted by vehicles, air conditioners, and the lack of vegetation, cities contained more heat and exhibited little latent heat of vaporization [3]. As a result, the UHI intensity tended to be much stronger at nighttime. However, some studies showed that the daytime UHI intensity was greater by using LST to characterize the SUHI [63]. In the daytime in our study, there were some unused land types consisting of bare soil and saline land in the northwest part, the high temperatures of which made the UHI intensity not that severe in the late morning to noon (from 08:00 to 12:00). 

**Figure 11:**
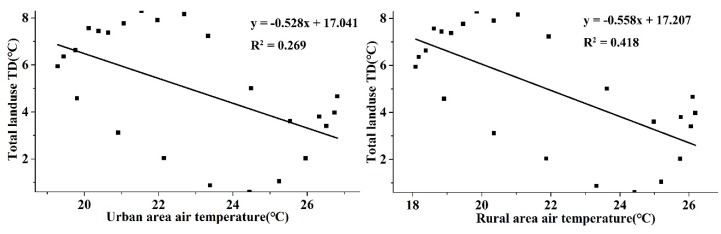
Correlations between total land use TD and urban and, rural temperatures.

Figure 11 shows that the total land use TD presented negative correlations with both urban and rural temperatures. However, the influence of rural temperature on the total land use TD was stronger than that of the urban temperature. It seemed that with the increase in temperature, the temperature difference between land use types became smaller.

### 4.4. Limitations

It should be noted that there may have been some limitations in this study. Although there were enough weather stations in this study, the interpolation maps may have yielded some inaccurate results. There were no weather stations in or near the water area. As a result, the temperature of the water body may not have been accurately modelled or represented. There are many factors that can affect the thermal environment, and more factors should be considered in future studies, such as the differences among rainy days, cloudy days, and days with gale weather. In addition, more detailed urban information should be considered to determine the variation of UHI intensity, for example, the use of local climate zone could be a good choice. Therefore, traditional observations with new technology, such as high spatial resolution remote sensing and thermal infrared remote sensing may provide a more comprehensive understanding of the mechanism of the UHI effect with efforts from different fields.

## 5. Conclusions

Hourly air temperature data collected from 195 meteorological stations during August 1–31, 2015 in Changchun, which is a city located in a cold zone, were used to characterize UHI intensity and the thermal environment. Based on these data, this study examined the hourly evolution of air temperature, UHI intensity and the relationships between the thermal environment and spatial pattern of land use types using various methods:

(1) The hourly trend of UHI intensity in the summertime was very different from that of air temperature, and looked like an elongated” V”. High air temperature did not indicate severe UHI intensity. There was about a 40% possibility of “inverse UHI intensity” from 08:00 to 13:00.
(2) The spatial pattern of the thermal environment varied significantly in different areas and at different hours. For temperature differences among land use types, paddy land was the coolest in the daytime, while woodland had the lowest temperature during the nighttime and the largest TDDN. It appeared that 23°C was the threshold across which land use was not sensitive to high temperatures.
(3) The differences in the cooling and warming rates among different land use types made the UHI intensity decline sharply after sunrise and increase quickly after sunset. The total land use temperature difference showed negative relationships with both urban and rural temperatures.

## Figures and Tables

**Table 1:** The classification of air temperature (AT) zones for the whole study area.

AT Zones	AT Range
lowest	AT < AT_mean_-1.5S
lower	AT_mean_ -1.5S ≤ AT < AT_mean_ − 1.0S
low	AT_mean_ -1.0S ≤ AT < AT_mean_ − 0.5S
medium	AT_mean_ -0.5S ≤ AT < AT_mean_ + 0.5S
high	AT_mean_ +0.5S ≤ AT < AT_mean_ + 1.0S
higher	AT_mean_ +1.0S ≤ AT < AT_mean_ + 1.5S
highest	AT ≥ AT_mean_ +1.5S

where *AT_mean_* is the mean air temperature of the study area and S is the standard deviation of AT.

**Table 2:** Area (km^2^) and percentage (%) of different land use types for the study area.

Land Use	Urban Area	Dry Land	Paddy Land	Wood Land	Rural Settlements	Other Built-Up Area	Unused Land	Water	Total
Area	577.5	14,250.4	1946.1	1475.4	1556.6	115.9	315.1	356.4	20,593.5
Percentage	2.80	69.20	9.45	7.16	7.56	0.56	1.53	1.73	100

**Table 3:** Urban, rural air temperatures (AT) (℃) for the period of August 1–31, 2015 for 195 stations.

	Maximum Hourly		Minimum Hourly		Daytime		Night		Mean Hourly	
	Urban	Rural	Urban	Rural	Urban	Rural	Urban	Rural	Urban	Rural
AT	26.81	26.18	19.27	18.08	24.03	23.49	21.09	19.58	22.85	21.93
SD	3.89	3.83	2.48	2.14	3.21	3.03	2.68	2.25	2.94	2.65
Time	15:00	14:00	05:00	05:00	05:00-18:00		19:00–04:00		00:00–23:00	

where SD is standard deviation of AT.

**Table 4:** The mean hourly UHI intensity (°C) of the study area for the period of August 1–31, 2015.

	Maximum	Minimum	Daytime	Nighttime	Mean
UHI intensity	1.64	0.03	0.49	1.51	0.92
standard deviation	1.01	0.75	0.65	0.82	0.62
Time	20:00	10:00	05:00–18:00	19:00–04:00	00:00–23:00

**Table 5:** The proportion (%) of different temperature zones and the H-region according to the mean hourly temperatures for Changchun during August 1–31, 2015. The largest and smallest proportions are highlighted with dark and light gray shadows, respectively.

Time	Lowest	Lower	Low	Medium	High	Higher	Highest	H-Region
00:00	5.42	7.77	18.47	44.26	13.82	4.54	5.73	24.09
01:00	5.24	6.49	18.34	47.89	11.75	4.12	6.17	22.04
02:00	4.08	7.48	15.31	51.24	11.09	4.20	6.60	21.89
03:00	3.74	7.54	16.19	50.10	11.69	4.33	6.42	22.44
04:00	3.73	8.30	15.42	47.56	14.05	4.19	6.76	25
05:00	3.72	7.48	16.27	51.85	10.38	3.95	6.35	20.68
06:00	5.59	6.29	15.14	52.50	10.85	3.55	6.08	20.48
07:00	7.63	5.69	10.52	46.51	15.21	8.84	5.60	29.65
08:00	9.27	8.26	8.45	42.25	16.59	10.80	4.36	31.75
09:00	8.86	8.95	10.48	39.53	19.58	8.68	3.92	32.18
10:00	4.74	10.30	13.64	40.64	17.06	8.80	4.82	30.68
11:00	5.52	11.71	13.72	31.22	21.84	11.84	4.13	37.81
12:00	5.51	11.65	15.12	35.46	14.52	11.90	5.83	32.25
13:00	4.78	11.86	16.75	32.13	14.57	13.46	6.44	34.47
14:00	5.16	13.90	16.47	28.44	17.61	12.05	6.36	36.02
15:00	6.53	5.92	22.78	28.38	22.60	8.90	4.89	36.39
16:00	6.10	11.30	16.78	25.28	25.51	11.33	3.70	40.54
17:00	6.97	7.90	17.01	31.74	20.94	11.05	4.39	36.38
18:00	6.16	8.56	15.89	37.35	17.61	8.42	6.01	32.04
19:00	4.88	10.43	16.80	37.31	17.82	6.75	6.01	30.58
20:00	4.52	12.07	17.68	40.35	13.90	5.46	6.02	25.38
21:00	4.74	12.09	16.04	39.39	16.70	5.51	5.53	27.74
22:00	4.10	10.75	18.23	43.19	13.18	4.64	5.91	23.73
23:00	4.35	10.42	17.76	40.91	15.95	4.92	5.69	26.56

**Table 6:** Temperature at different times for different land use types (°C). The highest and lowest temperatures are highlighted with dark and light gray shadows, respectively.

Time	Water Land	Unused Land	Paddy Land	Dry Land	Woodland	Urban Area	Rural Settlements	Other Built-Up Area
00:00	19.19 ± 0.41	19.30 ± 0.32	19.06 ± 0.46	19.10 ± 0.40	18.91 ± 0.42	20.20 ± 0.74	19.14 ± 0.43	19.37 ± 0.60
01:00	18.94 ± 0.44	18.99 ± 0.33	18.78 ± 0.43	18.81 ± 0.38	18.66 ± 0.41	19.94 ± 0.74	18.85 ± 0.41	19.10 ± 0.60
02:00	18.72 ± 0.45	18.71 ± 0.31	18.54 ± 0.42	18.57 ± 0.38	18.43 ± 0.40	19.72 ± 0.73	18.62 ± 0.41	18.90 ± 0.60
03:00	18.51 ± 0.41	18.48 ± 0.28	18.38 ± 0.37	18.37 ± 0.34	18.25 ± 0.37	19.38 ± 0.64	18.41 ± 0.36	18.65 ± 0.51
04:00	18.31 ± 0.38	18.22 ± 0.26	18.21 ± 0.35	18.18 ± 0.33	18.06 ± 0.38	19.14 ± 0.63	18.21 ± 0.35	18.43 ± 0.50
05:00	18.22 ± 0.37	18.13 ± 0.24	18.16 ± 0.32	18.10 ± 0.32	17.99 ± 0.36	19.00 ± 0.59	18.12 ± 0.33	18.31 ± 0.47
06:00	18.96 ± 0.32	19.03 ± 0.21	18.98 ± 0.34	18.95 ± 0.31	18.83 ± 0.35	19.62 ± 0.46	18.95 ± 0.32	19.06 ± 0.41
07:00	20.27 ± 0.32	20.52 ± 0.28	20.30 ± 0.35	20.37 ± 0.32	20.24 ± 0.36	20.80 ± 0.32	20.36 ± 0.34	20.41 ± 0.39
08:00	21.73 ± 0.38	22.00 ± 0.32	21.79 ± 0.45	21.89 ± 0.38	21.66 ± 0.40	22.12 ± 0.23	21.88 ± 0.41	21.86 ± 0.40
09:00	23.18 ± 0.45	23.44 ± 0.32	23.27 ± 0.47	23.34 ± 0.38	23.09 ± 0.39	23.40 ± 0.20	23.33 ± 0.41	23.26 ± 0.36
10:00	24.27 ± 0.39	24.60 ± 0.32	24.38 ± 0.37	24.44 ± 0.35	24.21 ± 0.35	24.48 ± 0.19	24.44 ± 0.37	24.44 ± 0.31
11:00	25.06 ± 0.36	25.44 ± 0.34	25.06 ± 0.42	25.20 ± 0.37	25.00 ± 0.36	25.32 ± 0.24	25.21 ± 0.39	25.24 ± 0.35
12:00	25.60 ± 0.36	26.07 ± 0.32	25.52 ± 0.40	25.71 ± 0.42	25.59 ± 0.37	26.02 ± 0.29	25.74 ± 0.42	25.87 ± 0.38
13:00	25.88 ± 0.54	26.52 ± 0.35	25.69 ± 0.48	25.98 ± 0.55	25.95 ± 0.46	26.54 ± 0.42	26.03 ± 0.55	26.35 ± 0.52
14:00	26.05 ± 0.62	26.67 ± 0.36	25.80 ± 0.55	26.08 ± 0.56	26.08 ± 0.46	26.77 ± 0.47	26.15 ± 0.57	26.57 ± 0.59
15:00	25.99 ± 0.58	26.49 ± 0.31	25.79 ± 0.56	26.02 ± 0.51	26.01 ± 0.45	26.80 ± 0.42	26.09 ± 0.52	26.51 ± 0.51
16:00	25.65 ± 0.52	26.05 ± 0.27	25.51 ± 0.57	25.68 ± 0.50	25.68 ± 0.44	26.31 ± 0.34	25.75 ± 0.50	26.07 ± 0.39
17:00	24.89 ± 0.52	25.26 ± 0.27	24.78 ± 0.53	24.91 ± 0.47	24.90 ± 0.41	25.52 ± 0.32	24.97 ± 0.48	25.29 ± 0.40
18:00	23.55 ± 0.47	23.91 ± 0.28	23.44 ± 0.51	23.56 ± 0.45	23.49 ± 0.40	24.37 ± 0.44	23.62 ± 0.46	23.98 ± 0.48
19:00	21.92 ± 0.44	22.25 ± 0.34	21.80 ± 0.54	21.88 ± 0.47	21.72 ± 0.43	23.00 ± 0.67	21.94 ± 0.49	22.27 ± 0.64
20:00	21.09 ± 0.50	21.33 ± 0.36	20.95 ± 0.56	21.00 ± 0.49	20.79 ± 0.47	22.25 ± 0.79	21.07 ± 0.52	21.37 ± 0.72
21:00	20.42 ± 0.44	20.62 ± 0.34	20.31 ± 0.53	20.33 ± 0.46	20.10 ± 0.46	21.53 ± 0.80	20.39 ± 0.49	20.65 ± 0.68
22:00	19.94 ± 0.46	20.08 ± 0.33	19.83 ± 0.51	19.84 ± 0.45	19.60 ± 0.47	21.09 ± 0.82	19.89 ± 0.47	20.17 ± 0.69
23:00	19.54 ± 0.43	19.67 ± 0.32	19.41 ± 0.47	19.45 ± 0.42	19.24 ± 0.45	20.62 ± 0.77	19.50 ± 0.44	19.75 ± 0.62
Mean	21.91 ± 2.90	22.16 ± 3.10	21.82 ± 2.90	21.91 ± 2.98	21.77 ± 3.02	22.66 ± 2.76	21.94 ± 2.98	22.16 ± 3.00
Nighttime	19.66 ± 1.18	19.77 ± 1.30	19.53 ± 1.18	19.55 ± 1.21	19.38 ± 1.18	20.69 ± 1.26	19.60 ± 1.22	19.87 ± 1.25
Daytime	23.52 ± 2.69	23.87 ± 2.87	23.46 ± 2.63	23.59 ± 2.72	23.48 ± 2.76	24.08 ± 2.69	23.62 ± 2.74	23.80 ± 2.82
TDDN	3.86	4.10	3.93	4.03	4.10	3.38	4.01	3.93
